# Me, myself and you: How self-consciousness influences time perception

**DOI:** 10.3758/s13414-023-02767-5

**Published:** 2023-08-10

**Authors:** Giovanna Mioni, Andrea Zangrossi, Sabrina Cipolletta

**Affiliations:** 1 Department of General Psychology, University of Padova, Via Venezia, 8, 35131 Padova, Italy; 2 Padova Neuroscience Center (PNC), University of Padova, Padova, Italy

**Keywords:** Self-consciousness, Social stimuli, Time processing, Time reproduction

## Abstract

Several investigations have shown that the processing of self-relevant information differs from processing objective information. The present study aimed to investigate the effect of social stimuli on subjective time processing. Here, social stimuli are images of an unknown male and female person and an image of participants’ self. Forty university students were tested with a time reproduction task in which they were asked to reproduce the duration of the stimulus previously presented. Images of others or themselves were used to mark the temporal intervals. Participants also performed questionnaires to evaluate the level of anxiety and depression as well as self-consciousness. A generalised linear mixed-effects model approach was adopted. Results showed that male participants with higher Private Self-Consciousness scores showed higher time perception accuracy than females. Also, female participants reported higher scores for the Public Self-Consciousness subscale than male participants. The findings are discussed in terms of social context models of how attention is solicited and arousal is generated by social stimuli, highlighting the effect of social context on subjective perception of time.

## Introduction

We constantly experience the flow of time, but the exact nature of time is not well understood. Psychological time serves several important functions that are essential for being able to act and survive in a dynamic environment. Our perception of time is relative and influenced by many different features; indeed, the literature on timing contains many examples showing that subjective time is greatly modulated by stimulus characteristics and context (Droit-Volet & Meck, 2007; Lake et al., 2016). Subjective time is not isomorphic to physical time: an hour may pass painfully slowly while waiting for a phone call from a loved one. The same duration, when we are entertained, may elapse hardly noticed. In waiting situations, but especially when we are bored, we feel trapped in time (Wittmann, 2015; Zakay, 2014).

These common experiences can be explained by variations in the sense of self. In waiting situations without the possibility of distraction, when time is in the focus of awareness and duration expands, self-consciousness is most pronounced. In contrast, when we are absorbed in a pleasant activity, we are less aware of ourselves. Time is hardly noticed and therefore contracts. These everyday experiences are in line with a theoretical framework of self-regulation where effortful emotion regulation accompanied by an increased self-awareness elongates the felt duration (Vohs & Schmeichel, 2003). Self-regulation is understood to occur when the self undergoes adaptations by resisting temptation and inhibiting behaviour, i.e., by altering one’s mood when staying attentive during a boring lecture; all this is done by executive aspects of the self (Baumeister & Vohs, 2003). Self-regulation also refers to positive emotions such as the feeling of awe, which evokes an update of one’s mental schema and leads to the feeling of time expansion (Rudd et al., 2012). In contrast, when mind wandering or daydreaming, we lose touch with the present state of ourselves and of time; duration is then relatively underestimated (Terhune et al., 2014).

Cognitive models have attempted to explain temporal processing, such as the Attentional Gate Model (Zakay & Block, 1995) – when attention is not fully allocated to time less temporal information is stored in the accumulator and the temporal interval is more likely to be judged as short. An attentional gate is placed before the accumulator, which controls the flow of pulses from the pacemaker to the accumulator. In an uninspired waiting situation, we focus more on time, leading to more accumulated pulses and a corresponding relative overestimation; whereas when we are distracted by activities we focus less on time, thus evoking relative time contraction through a smaller amount of represented time units (Zakay & Block, 1995).

In comparing the model of self-regulation and the attentional-gate model one is tempted to conclude that time consciousness and self-consciousness are strongly related and thus are jointly modulated in a situation when attention is actually directed to time: if the experience of the self is intensified, the subjective feeling of time changes accordingly, and is experienced as expansion or as slowing down of the passage of time.

Profound individual differences can be observed in self-awareness, some persons constantly think about themselves, monitor their behaviour, and ruminate over their thoughts. At the other extreme are persons whose absence of self-consciousness is so complete that they have no understanding of either their own motives or of how they appear to others. Several investigations have shown that the processing of self-relevant information differs from processing other information. Ainley and colleagues (2012) demonstrated that mirror self-observation (which relies on exteroception) during an interoceptive task enhanced subjective interoceptive awareness in participants with low interoceptive awareness. Individuals with low baseline interoceptive awareness showed significant increases in accuracy when performing the heartbeat detection task whilst looking at their face in a mirror as compared to looking at a black screen. During mirror self-observation, the perception of one’s own face may evoke an integrated self-awareness, which then enhances processing of other self-related bodily information, such as interoception, via a top-down gating of attention. Maister and Tsakiris (2014) step forward into this investigation looking at cultural differences (Western and Asian cultures). Participants with poor interoceptive awareness showed an improvement during self-face observation, but not during other-face observation. However, in the East Asian group, no significant changes in interoceptive awareness were observed in either face condition. Ainley et al. (2012) proposed that the exteroceptive perception of one’s own face may facilitate the processing of other self-related bodily information, such as interoceptive signals, via a process of attentional gating. This interaction between interoceptive and exteroceptive systems suggests that the exteroceptive perception of one’s body, such as during self-face observation, can evoke an integrated bodily self-awareness.

Within the context of temporal processing, Effron and colleagues (2006) showed that imitation of the expression must occur to influence temporal perception; if imitation of the facial expressions was prevented, no modification on perceived duration was found. Chambon and colleagues (2008) showed that the presentation of older faces leads to more short responses than the presentation of younger faces, but this effect occurred only when the participant’s and stimulus faces were of the same sex (Chambon et al., 2008). That being said, Kliegl et al. (2015) failed to reproduce such an interaction of sex concordance when studying the effect of angry faces on time perception. However, they showed that the presentation duration of faces of the opposite sex tends to be overestimated compared to that of faces of the same sex. Finally, Grondin et al. (2015) observed an effect of sex on perceived duration indicating that female participants overestimated time when male angry faces were used. Moreover, the emotional effects on the participants’ performance were correlated to empathic abilities. Indeed, the effects of emotion on time perception depend on the observers’ attributes such as their sex and their empathic abilities, and this effect is stronger for female than for male participants.

The present study aimed to investigate the effect of self-consciousness on subjective time processing. Here, social stimuli are images of an unknown male and female person and an image of the participant’s self. In particular, we focused on the effect of other versus self-images on the subjective time processing by using a time reproduction task. A temporal reproduction task involves presenting participants with a given time interval (encoding phase) and asking them to reproduce this interval (reproduction phase). Social stimuli will be presented in the encoding phase and used as a marker for the temporal intervals. We predict that participants with a higher level of Private Self-Consciousness are more attracted by the presentation of their own face, resulting in lower temporal abilities. According to the Attentional Gate Model (Zakay & Block, 1995), if attention is not fully allocated to time less temporal information is stored, resulting in lower accuracy and higher variability. We also predict a higher level of temporal accuracy in participants with a higher level of Public Self-Consciousness consistent with the idea that these individuals have a greater desire in achieving good performance (e.g., sports or school; Baumeister, 1984; Hatzigeorgiadis, 2002; Martin & Debus, 1998).

## Method

### Participants

Forty participants were recruited and tested at the Department of General Psychology, University of Padova. All participants were university students; 20 were male (mean age = 24.25 years, *SD* = 2.90) and 20 were female (mean age = 24.00 years, *SD* = 1.65).

To test whether our sample size allowed us to reach an acceptable statistical power, we ran a power analysis through simulation by using the R package simr (Green & MacLeod, 2016), setting a 5% chance of a Type I error (α = 0.05) and a 20% chance of a Type II error (β = 0.2), and we found that with our sample size we could reach a statistical power of 85%.

### Materials

#### Time reproduction task

Participants were instructed to reproduce the duration of a previously viewed stimulus. The stimulus (image; see *Procedure*) appeared at the centre of the computer screen for 1, 2 or 3 s. Images were presented during the encoding phase, and a grey oval was presented during the reproduction phase. After a 500-ms inter-stimulus interval, a question mark appeared on the computer screen and participants were instructed to press the spacebar for the same duration that the stimulus was on the screen. Participants were instructed to refrain from counting (Rattat, & Droit-Volet, 2012). Participants performed three blocks; each duration was randomly presented six times in each block for a total of 54 trials.

#### Questionnaires

The *State-Trait Anxiety Inventory Scale* (STAI; Spielberger et al., 1983; Italian adaptation by Pedrabissi & Santinello, 1989; cut-off score 51 for men and 58 for women) was used to assess anxiety state (STAI-Y1) and trait (STAI-Y2) and the *Beck Depression Inventory* (BDI-II; Beck et al., 1996; Italian adaptation by Ghisi et al., 2006) was used to assess level of depression (0–9 indicates minimal depression, 10–18 indicates mild depression, 19–29 indicates moderate depression, and 30–63 indicates severe depression).^1^ The *Self-Consciousness Scale* (SCS; Scheier & Carver, 2013) measures how people reflect on themselves, and includes 21 items; higher scores indicate a higher level of self-consciousness. Participants are instructed to respond how much each statement reflects him-/herself by indicating on a 4-point Likert scale from 1 = “a lot like me” to 4 = “not like me at all”. The SCS includes three sub-scales: “Private Self-Consciousness” (I'm always trying to figure myself out), “Public Self-Consciousness” (“I'm concerned about my style of doing things”), and “Social Anxiety” (“It takes me time to get over my shyness in new situations”). Private self-consciousness is a tendency to be introspective and examine one's inner self and feelings. Public self-consciousness is an awareness of the self as it is viewed by others. This kind of self-consciousness can result in self-monitoring and social anxiety. Both private and public self-consciousness are viewed as personality traits that are relatively stable over time (Martin & Debus, 1999; Scheier & Carver, 1985).

### Procedure

Participants were tested individually in a quiet room at the Department of General Psychology, University of Padova. Upon arrival at the lab, they were photographed (Nikon Coolpix 3,200 resolution of 3.2 megapixel). The photo was taken about 100 cm from the participant with a grey background similar to the one presented in the two images selected from FACES dataset (Ebner et al., 2010) and used as control images. From the FACES database, we selected a picture of a young female (048) and a young male (066). All participants performed the time reproduction task with these two control images; moreover, they performed the task with their own image as stimulus. While the experimenter selected participant’s image and included it in the experimental task, participants performed the questionnaires. The experimental procedure lasted approximately 60 min. The study was approved by the Ethics Committee of Psychological Research of the University of Padova, and all participants gave their written consent to participate in the study.

### Statistical analyses

The data were analysed in terms of the Absolute Discrepancy (AD), calculated by putting in absolute value the difference between the time reproduction (Rd) and the target duration (Td) [AD=|Rd – Td|] (Glicksohn & Hadad, 2011). A greater AD indicated lower performance since the time reproduced was farther from the target duration (Table 1). Independent *t*-test analyses were computed on STAI-Y1, STAI-Y2, BDI-II, and SCS questionnaires.

**Table 1:** Mean and standard deviation of raw data and absolute discrepancy

		Raw (ms)			Absolute discrepancy (ms)		
		Same sex	Opposite sex	Self	Same sex	Opposite sex	Self
Female	1000	898 (217)	869 (252)	906 (247)	206 (115)	240 (143)	222 (136)
	2000	1577 (228)	1522 (175)	1625 (232)	429 (217)	477 (175)	386 (213)
	3000	2203 (267)	2229 (354)	2269 (335)	797 (267)	771 (355)	731 (334)
Male	1000	978 (199)	991 (210)	937 (226)	152 (124)	166 (123)	183 (140)
	2000	1566 (188)	1665 (237)	1715 (286)	433 (188)	352 (208)	360 (174)
	3000	2223 (316)	2313 (333)	2291 (341)	776 (316)	687 (333)	709 (341)

A generalised linear mixed-effects model approach (Pinheiro & Bates, 2000) was adopted to investigate the effects of sex, stimulus condition (self, same sex, opposite sex), and SCS subscales (i.e., Private Self-Consciousness, Public Self-Consciousness, or Social Anxiety) on AD. This statistical approach has recently been applied to many research areas as it gives the opportunity to consider both variables under experimental control (i.e., fixed-effects) and factors not directly controllable by the experimenter, the so-called random-effects factors (e.g., participants).

In the present study, we wanted to highlight the most plausible model (i.e., set of predictors) given the AD data. Therefore, we built three sets of nested linear Mixed-Effects regression models, sharing a common structure: AD as dependent variable and participant as a random-effect factor. Then, each set considered one among three SCS subscales (Private Self-Consciousness, Public Self-Consciousness or Social Anxiety), and each model was built with a different combination of sex, stimulus condition, SCS subscale or their interactions as fixed-effect factors. Thus, a total of 18 models were built, including three models that were shared between the three sets (M0 – the null model: considering the random-effect only; M1 – including sex only, and M2 – including sex + stimulus condition as predictors), and five models (M3–M7) for each SCS subscale (i.e., with a combination of predictors including one SCS subscale).

All models were fit using the maximum likelihood estimation (ML) and a Likelihood-Ratio Test (LR) was performed to compare each model with the previous one (i.e., including all predictors but one). This test is adopted to determine if there are significant differences between models with respect to their goodness-of-fit weighted for the number of predictors, i.e., the trade-off between explained variance and degrees of freedom.

**Fig. 1:**
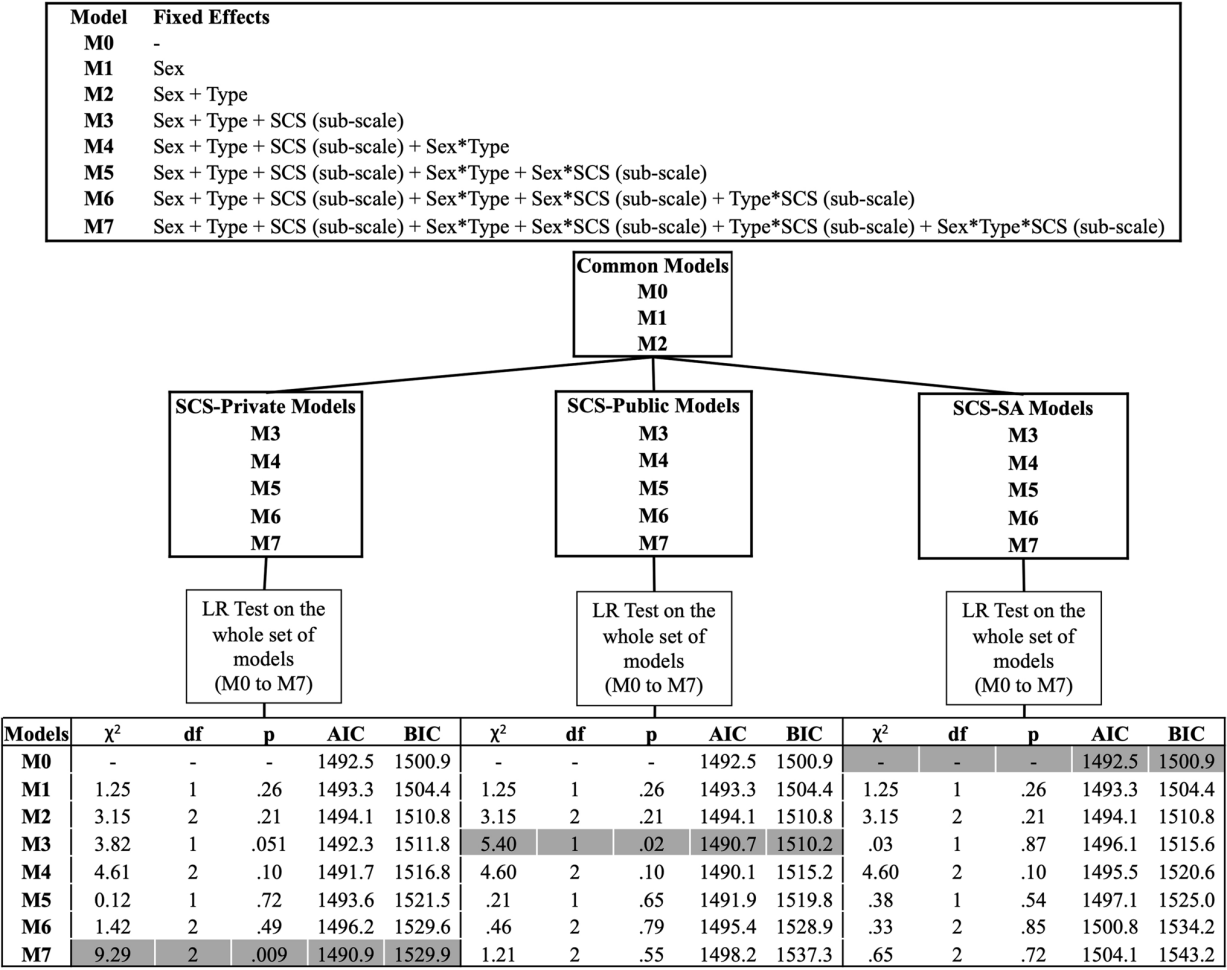
Graphical representation of all built models reporting the fixed-effect factors included in each one, and the model selection procedure. Rows highlighted in grey indicate the best model for each comparison

Moreover, the contribution of each model toward explaining data was measured by means of two indices, namely the Akaike Information Criterion (AIC; Akaike, 1998) and the Bayesian Information Criterion (Schwarz, 1978)﻿. Comparing AIC and BIC through different models allowed us to give further support to our selection by taking into consideration model parsimony and quantifying the increase in model fit weighted by the number of involved predictors. This procedure was repeated for each set of models. Notably, cases of discrepancy between AIC and BIC occur when the model with the highest likelihood was not that with the lowest number of predictors. In such cases we considered AIC as this index tends to favour model fit, as compared to BIC. Figure 1 shows a graphical representation of all built models reporting the fixed-effect factors included in each one, as well as the results of models’ comparison (the best model is highlighted).

We also ran a sensitivity analysis to check the robustness of our model comparison and to highlight observations potentially influencing model fit. Thus, the procedure described above was repeated N times (N=40), each time excluding data from one subject from the analysis, and the consistency of the results (i.e., the best model selected) was tested. For all the sets of models, the sensitivity analysis confirmed the best model highlighted using the full sample.

Furthermore, least-squares post hoc contrasts were performed to investigate significant interactions, in the best model for each set of predictors. P-values were corrected by means of the False Discovery Rate (FDR) to control for Type I error. All reported analyses were performed by means of R Software (version 4.2.1; R Core Team, 2016), and the R package lme4 (Bates et al., 2014) was used for generalised mixed-effects models.

## Results

### Questionnaires

Significant differences between male and female participants were observed for the Public Self-Consciousness [*t*(38) = 2.43, *p* = .020] subscale, indicating that female participants reported a higher level of public self-consciousness. No differences were observed between groups for Private Self-Consciousness or Social Anxiety (all *p* ≥ .232). See Table 2 for further details.

**Table 2:** Means and standard deviations for the measures used

	Male	Female	*t*
	M (*SD*)	M (*SD*)	
STAI-Y1	35.75 (8.74)	35.70 (9.53)	2.02
STAI-Y2	45.40 (9.17)	42.10 (11.28)	1.01
BDI-II	8.45 (5.85)	7.25 (7.81)	0.55
SCS – Total score	59.45 (4.54)	63.65 (5.61)	2.60*
SCS – Private Self-Consciousness	27.25 (3.14)	28.55 (3.60)	1.21
SCS – Public Self-Consciousness	18.85 (2.01)	20.80 (2.97)	2.43*
SCS – Social Anxiety	13.35 (3.26)	14.30 (4.00)	0.82

*STAI-Y1* State-Trait Anxiety Inventory Scale – state, *STAI-Y2* State-Trait Anxiety Inventory Scale – trait (Spielberger et al., 1983, Italian adaptation by Pedrabissi & Santinello, 1989), *BDI-II* Beck Depression Inventory (Beck et al., 1996, Italian adaptation by Ghisi et al., 2006), *SCS* Self-Consciusness Scale (Scheier & Carver, 2013)

* *p* < .05

### Self-Consciousness Scale (SCS)-Private subscale

As shown in Fig. 1 (bottom-left table), the LR test highlighted model M7 as the best model (χ^2^[2] = 9.29; *p* = .009; AIC = 1490.9; BIC = 1529.9), thus suggesting that the model fit to the data significantly benefitted from the adding of the three-way interaction between sex, stimulus condition, and SCS-Private score.

**Fig. 2:**
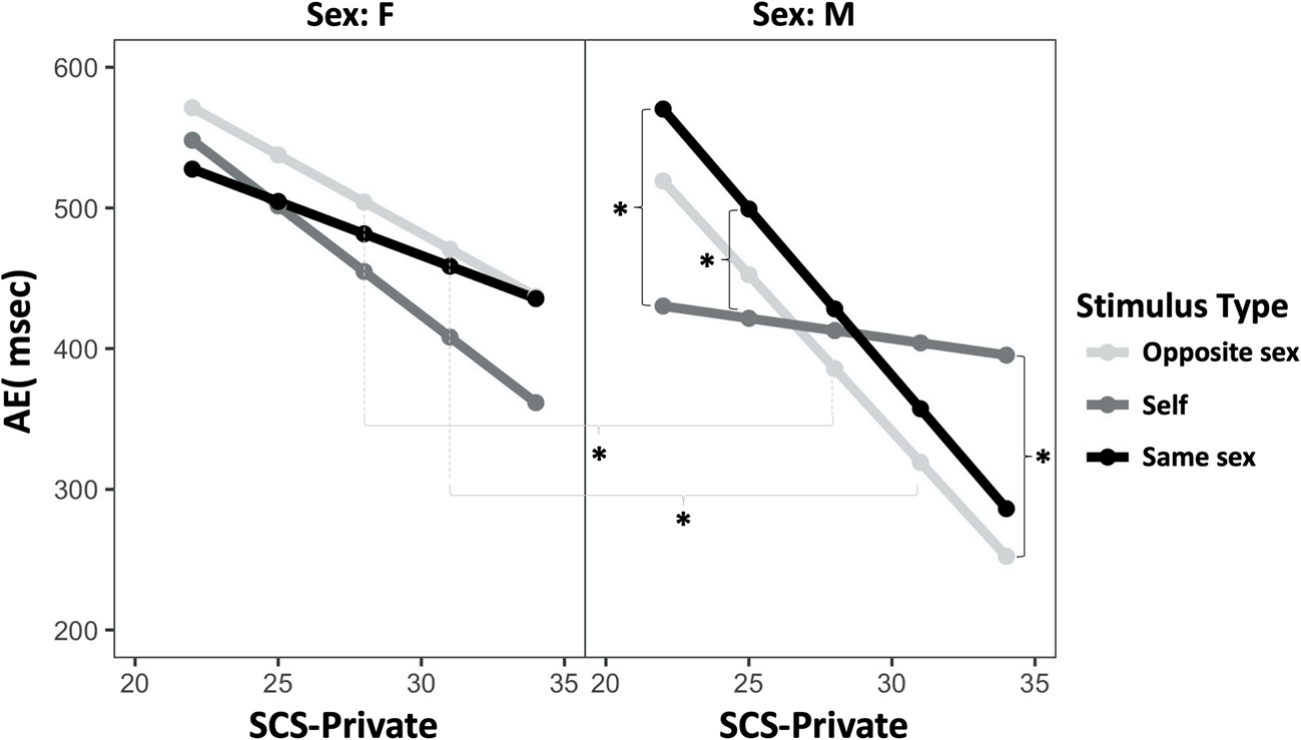
Mean Absolute Discrepancy (AD) in female and male participants as a function of stimulus condition (other, self and same) and Self-Consciousness Scale (SCS)-Private score

A Wald χ^2^ test was run to test the fixed effects and revealed a significant main effect of SCS-Private score (χ^2^[1] = 4.02; *p* = .045), suggesting that higher scores were associated with higher accuracy in the time perception task (i.e., lower AD). Moreover, the interaction sex * stimulus condition (χ^2^[2] = 6.04; *p* = .049) was significant, indicating that the effect of sex on AD was modulated by the stimulus condition. However, the three-way interaction between these effects (i.e., sex × stimulus condition × SCS-Private score) was also significant (χ^2^[2] = 9.85; *p* = .007; Fig. 2). This result suggests that the relation between the private component of self-consciousness and AD is subjected to the modulation of the interaction between the participant’s sex and stimulus condition. In other words, the improvement in time perception accuracy following SCS-Private score depended on the combination of a participant’s sex and the type of stimulus presented (i.e., whether it depicted the participant, a person with the same gender, or a person with another gender).

To further investigate this effect, we carried out post hoc contrasts using least-squares means. For this purpose, we fixed five levels of the SCS-Private subscale (i.e., 22, 25, 28, 31, 34) to compare the effect of sex and stimulus condition for different levels of the subscale. This analysis showed significant differences between males and females when stimuli of the opposite sex were involved for high values of the SCS-private subscale only (e.g., for SCS-private subscale = 28, *t* = 2.4, FDR-corrected *p* = .02).

On the other hand, contrasts carried out within each gender level revealed that males showed significantly higher AD for stimuli of the same sex compared to stimuli depicting themselves, but only for low levels of SCS-Private (i.e., SCS-private = 22, *t* = -2.97, FDR-corrected *p* = .01; SCS-private = 25, *t* = -2.6, FDR-corrected *p* = .03). Conversely, for high levels of the SCS-private subscale, males showed significantly lower AD values for stimuli of the opposite sex compared to stimuli depicting themselves (*t* = -2.49, FDR-corrected *p* = .04).

### SCS-Public subscale

The models’ comparison (LR test) carried out on the second set of models including SCS-Public subscale highlighted M3 (χ^2^[1] = 5.40; *p* = .02; AIC = 1490.7; BIC = 1510.2) as the best model (see Fig. 1, bottom-centre table). This suggests that adding the SCS-Public score to a model including only participant’s sex and stimulus condition (i.e., M2) significantly improved the model fit, and that the addition of further variables was not justified by a significant improvement in the goodness of fit.

**Fig. 3:**
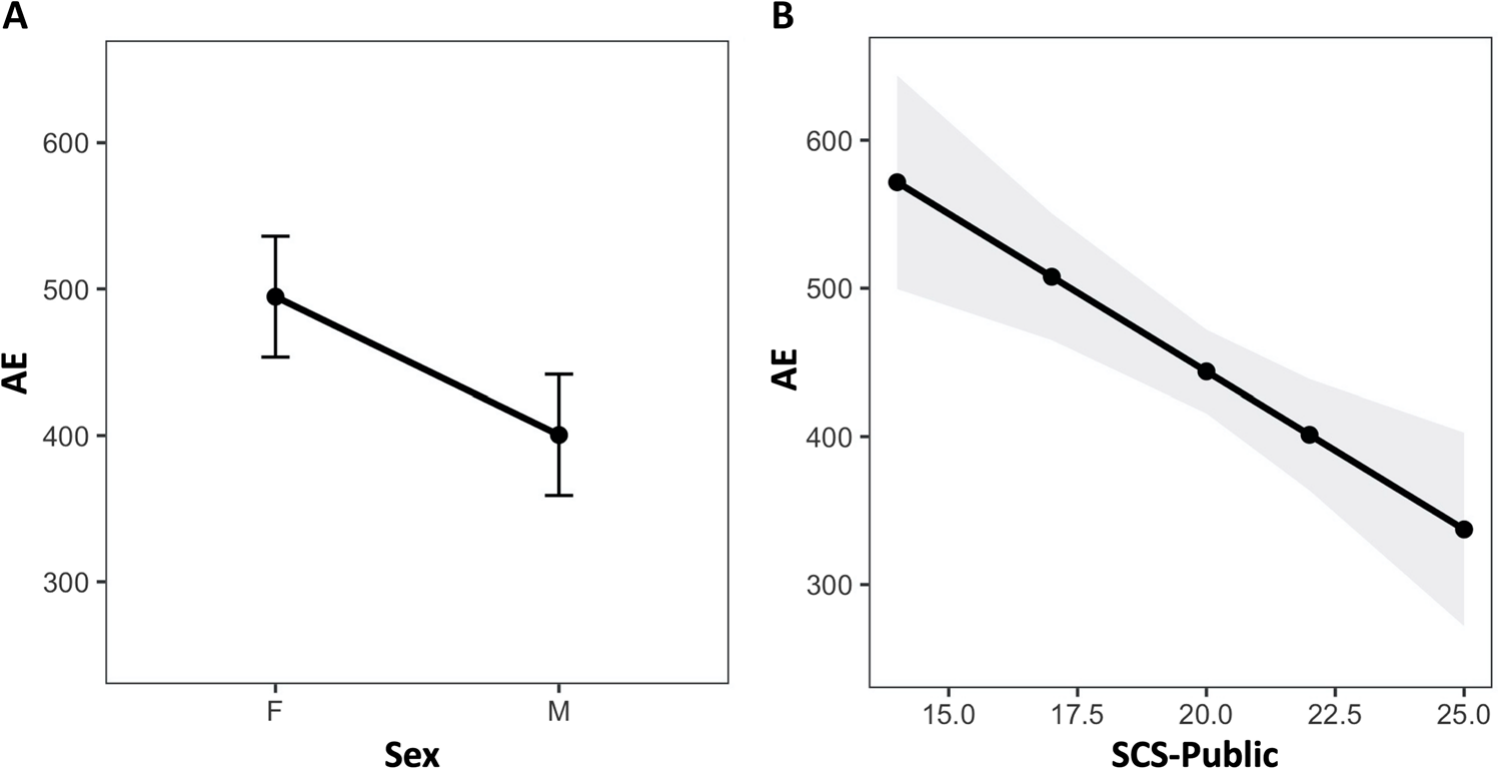
Mean Absolute Discrepancy (AD) (**A**) as a function of sex and (**B**) as a function of sex and Self-Consciousness Score (SCS)-Public score. Error bars and grey area represent the 95% confidence interval

Thus, we gave a deeper look into the M3 model fixed effects, which revealed a significant main effect of SCS-Public score on AD (χ^2^[1] = 5.78; *p* = .016; Fig. 3 left). This indicates that higher scores in this SCS subscale were associated with more accurate time perception performances (i.e., lower AD) in our sample.

Moreover, sex (χ^2^[1] = 4.02; *p* = .045) turned out to have a significant effect on AD (Fig. 3 right), suggesting an overall smaller AD for male compared to female participants (standardized-ß=-.29, 95% confidence interval (CI) = [-.57, -.006]). In other words, male participants seemed to show a more accurate performance in the time perception task.

### SCS-Social Anxiety subscale

When focusing on the third set of models (i.e., those including the SCS-Social Anxiety subscale as a measure of self-consciousness), the LR test suggested that any of the models from M1 to M7 were significantly better than the null model (M0), including the grand mean only (Fig. 1, bottom-right table). This indicates that the addition of further variables to M0 would not lead to a significant improvement in model fit. Moreover, this result suggests a weak effect of SCS-Social Anxiety subscale on AD. To support this result, we ran models from M3 (i.e., the simplest model including SCS-social Anxiety) to M7, and none of them showed a significant effect of this SCS subscale.

## Discussion

The ability to accurately estimate the passage of time plays an important role in daily activities; therefore, it is important to understand what factors are critical in determining our ability to correctly assess the passage of time. Even if adults are able to accurately estimate the passage of time, our perception of it is influenced by many different features. Indeed, the literature on time perception contains various examples showing that subjective time is greatly modulated by stimulus characteristics and context. For example, several studies have demonstrated the impact of emotional stimuli on time perception (Droit-Volet et al., 2013; Gil & Droit-Volet, 2011; Grondin, 2010; Lake, 2016). In particular, emotional facial expressions have been extensively used (Droit-Volet, 2014; Droit-Volet & Meck, 2007; Droit-Volet & Gil, 2009; Droit-Volet et al., 2013).

In an age of ‘selfies’, how we look at our own face assumes critical importance. Looking at a self-face is associated with greater attention to and faster recall compared to other faces (Devue et al., 2009; Tong & Nakayama, 1999). Identification of a self-face requires orientation toward the self from a decentralized position and indicates high salience for self-related stimuli (Heinisch et al., 2011). The self-face is identified faster among other faces even where faces are presented in non-upright conditions (Devue et al., 2009; Tong & Nakayama, 1999). Such high salience for self-related stimuli is also evident from their facilitatory effect on spatial priming (Pannese & Hirsch, 2011) and interference with cognitive tasks (Brédart et al., 2006).

The present study was designed to further investigate the effect of social stimuli defined as images of unknown males and females and an image of themselves on the subjective perception of time. In particular, we focused on the effect of other versus self-images in relation to the gender of participants. We predicted lower temporal abilities in our participants when faces of others were presented compared to self-images, consistent with the idea that images of ourselves require less attentional resources to be processed compared to images of an unknown person. Moreover, we predicted that the effect of images of an unknown person or of a participant’s self on time will also be influenced by the participant’s gender and by the gender of the unknown person (male vs. female) used to mark time. Indeed, Chambon and colleagues (2008) showed that the presentation of older faces leads to shorter responses than the presentation of younger faces, but only when the participant’s and stimulus faces were of the same sex.

Our results showed that participants differed in the level of the Public Self-Consciousness subscale, with female reporting higher scores compared to male participants. Our results are in line with Gould (1987), indicating a higher public self in female participants compared to male counterparts and similar levels in the Private and Social Anxiety subscales. It is possible that women have a greater ability to manage their roles in society with greater attention to themselves as social actors in social contexts. Participants with a higher level of Public Self-Consciousness also reported lower AD, indicating greater accuracy compared to participants with a lower level of Public Self-Consciousness. Participants with higher scores in this subscale are more prone to think about those aspects of self that are important for public display; this denotes attention to overt aspects of the self (e.g., physical appearance and overt behaviours) that others can observe as well as evaluate. Previous studies conducted to investigate the impact of self-evaluation on academic achievement reported that individuals with higher self-evaluation are more motivated to do school work and are more active in responding to perceived failure (Martin & Debus, 1998). Also, it has been reported that a higher score in public self-consciousness increases one’s sensitivity to pressure situations such as a sports competition (Baumeister, 1984; Hatzigeorgiadis, 2002). Given that public self-consciousness is related to self-presentation, it is therefore possible that individuals, when interacting with others (e.g., experimental setting, exams), attempt to present as favourable an image as possible with greater interest in achieving greater performance.

More interesting are the results concerning temporal performance in relation to individuals’ level of Private Self-Consciousness. The results indicate that male participants showed a higher accuracy for stimuli depicting participants themselves compared to stimuli of the same sex (and not for stimuli of the opposite sex), but only for lower levels of Private Self-Consciousness. Conversely, for higher private self-consciousness levels, male participants seemed to be more accurate when responding to stimuli involving the opposite sex compared with stimuli depicting themselves. These findings suggest that the advantage of male participants in processing stimuli representing themselves in a time perception paradigm (in terms of higher accuracy) can be detected only at low levels of private self-consciousness, while participants with high private self-consciousness were more accurate for stimuli of the opposite sex. Finally, sex-related differences in time perception accuracy were found only for participants with high private self-consciousness.

Studies investigating own-face processing often report that viewing our own face arouses more attention than processing other faces (Brédart et al., 2006; Devue & Brédart, 2008). Indeed faces, in general, are particularly difficult to ignore because of their biological and social significance (Scott & Fava, 2013), and specifically our own face seems to attract more attention as revealed by behavioural responses (Palermo & Rhodes, 2007) or electrophysiological measures (Bola et al., 2021; Tacikowski & Nowicka, 2010). Linking these previous observations with our findings, it is possible that participants with higher private self-consciousness were particularly attracted by their own face, engaged more attention in processing their own images rather than processing time, leading to lower temporal accuracy. Conversely, participants with lower private self-consciousness were less distracted by the presentation of their own face and presented higher accuracy when their own face was presented compared to unknown faces.

Our work might suggest practical implications in terms of gender differences in self-awareness and social context. It is well known that social media are massively impacting our lives and the way we interact with friends and peers (Lee et al., 2011; Valkenburg, 2017). According to data from the National Adolescence Observatory (2017), 94% of Italian teenagers use the Internet to talk with friends, and more than half (54%) use it to check their social profile. It is also known that women are more aware on their ‘public self’ than men, and this has consequences in their response to social approval and to health issues (Cipolletta et al., 2020). Moreover, an increase in self-awareness and rumination also impacts health attitudes. Women tend to ruminate more than men, and starting in adolescence and continuing through adulthood, women are twice as likely as men to experience depression (Johnson & Whisman, 2013). Future studies might consider manipulating the interplay between self-awareness and emotional responses by acting at the level of subjective experience of time and the representation of self in space and time. Also, future studies might be conducted to explore implicit self-representation and self-consciousness using time perception tasks. For example, previous studies have used temporal tasks to investigate implicit sensitivity to emotional stimuli in Parkinson disease (Mioni et al., 2016, 2018) and autism spectrum disorder patients (Jones et al., 2017), indicating preserved implicit emotional recognition with important implications for understanding how emotions are processed. Also, Gagnon and colleagues (2018) used images of food (joyful, disgusting and neutral) to elicit emotions in women suffering from eating disorders. The authors showed that women with anorexia nervosa overestimated time compared to controls and to women with bulimia nervosa; this effect indicated that women with anorexia nervosa experienced an intense negative reaction in response to food presentation. It is possible that the presentation of food images increased the arousal level and activated the defensive system in women with anorexia nervosa, which increased the internal clock speed resulting in temporal overestimation.

Taken together, our results confirm previous findings showing the effect of social and emotional stimuli on the subjective experience of time (Droit-Volet & Meck, 2007; Gil & Droit-Volet, 2011), and extend previous results showing that our perception of time also depends on our self-consciousness. Male participants with higher Private Self-Consciousness scores showed higher time perception accuracy than females. Private self-consciousness reflects the tendency to introspection and to examining one's inner self and feelings, probably expressing a more precise dialog between inner perception of time and cortical (e.g., prefrontal areas, insula) and subcortical (e.g., amygdala, hypothalamus) brain structures via the vagus nerve (Thayer et al., 2012; Wittmann, 2013, 2015). Following this line, Cellini et al. (2015) showed that higher vagal control was associated with higher accuracy in time reproduction task. The authors suggested that individuals with higher HRV may better adapt to changes in the environmental demands and be better prepared to respond to different task demands.

Our results are also in line with neuroimaging studies showing overlapping brain activity during time perception and self-processing. Specifically, some studies show that the BOLD (blood-oxygen-level-dependent)-signal in the precuneus correlated with the time interval duration both in healthy controls (Jech et al., 2005) and in Parkinson’s disease patients (Dušek et al., 2012), a clinical condition usually related to altered time estimation performance (Mioni et al., 2016, 2018; Pastor et al., 1992). Precuneus activation has been related to self-processing (Cavanna & Trimble, 2006) and is part of the so-called default mode network (DMN), a resting-state brain network strongly related to self-referential thoughts (Raichle, 2015). This fits with the idea of a modulatory role of self-consciousness on time perception performance.

## Data Availability

The data that support the findings of this study are openly available (Mioni, G. (2022, June 27). Me, myself and you: How self-consciousness influences time perception) and can be retrieved from osf.io/wbtz3 and https://osf.io/4fshr/?view_only=cb0c1f670a1f4779ba571e39450f923a

## Footnotes

1 All participants fell within the normal range in terms of anxiety and depression; moreover, no differences between male and female participants were observed for STAI-Y1 [*t*(38) = 0.02, *p* = .986], STAI-Y2 [*t*(38) = 1.02, *p* = .316] and BDI-II [*t*(38) = 0.55, *p* = .586].
